# A retrospective analysis of High-Dose Interleukin-2 (HD IL-2) following Ipilimumab in metastatic melanoma

**DOI:** 10.1186/s40425-016-0155-8

**Published:** 2016-09-20

**Authors:** Elizabeth I. Buchbinder, Anasuya Gunturi, Jessica Perritt, Janice Dutcher, Sandra Aung, Howard L. Kaufman, Marc S. Ernstoff, Girald P. Miletello, Brendan D. Curti, Gregory A. Daniels, Sapna P. Patel, John M. Kirkwood, Sigrun Hallmeyer, Joseph I. Clark, Rene Gonzalez, John M. Richart, Joe Lutzky, Michael A. Morse, Ryan J. Sullivan, David F. McDermott

**Affiliations:** 1 Dana Farber Cancer Institute, Boston, MA USA; 2 The Cancer Center – Lowell General Hospital, Lowell, MA 01854 USA; 3 Prometheus Labs, 9410 Carroll Park Drive, San Diego, CA 92121 USA; 4 Cancer Research Foundation of New York, Chappaqua, NY USA; 5 Rutgers Robert Wood Johnson Medical School, New Brunswick, NJ USA; 6 Cleveland Clinic, Cleveland, OH USA; 7 Hematology/Oncology clinic, Baton Rouge, LA USA; 8 Providence Health & Services, Portland, OR USA; 9 Moores UCSD Cancer Center, La Jolla, CA USA; 10 The University of Texas MD Anderson Cancer Center, Houston, TX 77030 USA; 11 University of Pittsburgh School of Medicine, Pittsburgh, PA USA; 12 Oncology Specialists, SC, Park Ridge, IL USA; 13 Loyola Medicine, Maywood, IL USA; 14 University of Colorado, Aurora, CO USA; 15 Saint Louis University, Saint Louis, MO 63110 USA; 16 Mount Sinai Medical Center, Miami Beach, FL USA; 17 Duke, Durham, NC USA; 18 Massachusetts General Hospital, Boston, MA USA; 19 Beth Israel Deaconess Medical Center, Boston, MA USA

**Keywords:** Melanoma, Interleukin-2, Immune Checkpoint blockade, Ipilimumab

## Abstract

**Background:**

High dose interleukin-2 (HD IL-2) can induce durable responses in a subset of patients leading to long-term survival. Immune checkpoint blockade (ICB) has demonstrated similarly durable responses in a larger proportion of patients. However, not all patients respond to immune checkpoint blockade and subsequent therapeutic options need to be explored.

**Methods:**

The PROCLAIM database was queried for patients with metastatic melanoma who had received HD IL-2 after treatment with ipilimumab or without prior ICB. Patient characteristics, toxicity and efficacy were analyzed.

**Results:**

A total of 52 metastatic melanoma patients were treated with high dose IL-2 after ipilimumab and 276 patients were treated with high dose IL-2 without prior ICB. The overall response rate in the prior ipilimumab group was 21 % as compared to 12 % in the group that had not received prior ipilimumab. The median overall survival, measured from the initiation of HD IL-2 therapy, was 19.3 months in the prior ipilimumab group and 19.4 months in the no prior ICB group. Toxicities observed on HD IL-2 were relatively equivalent between the groups although there were cases of CTLA4 antibody-induced colitis reported after HD IL-2 treatment and a CTLA4 antibody-induced colitis related death.

**Conclusion:**

In this retrospective analysis HD IL-2 therapy displayed antitumor activity in melanoma patients who progressed following treatment with ipilimumab. Most HD IL-2 toxicity was not worsened by prior ipilimumab therapy except for one treatment related death from colitis. Care should be taken to avoid reactivation of CTLA4 antibody-induced colitis.

**Electronic supplementary material:**

The online version of this article (doi:10.1186/s40425-016-0155-8) contains supplementary material, which is available to authorized users.

## Background

Immunotherapy has become the backbone of melanoma therapy and is rapidly expanding its role in renal cell carcinoma, lung cancer and other malignancies. Since 2011, three novel immune checkpoint blockade agents have been approved for the treatment of advanced melanoma and are recommended among the first line options for treatment [1–5]. However, there remain patients who do not respond to front line immune checkpoint blockade who require further treatment. For several decades, cytokine based immunotherapies such as high dose interleukin-2 (HD IL-2) have been shown to induce durable tumor responses in a subset of patients leading to long-term survival [6]. However, the toxicity profile of HD IL-2 is high which limits its application [7].

Regulation of the immune system is complex and involves a number of regulatory checkpoints, growth factors, cytokines and regulatory immune cells that play a role in host anti-tumor immunity. A key observation in the clinic is that lack of response to one immune checkpoint inhibitor class such as CTLA-4 blockade does not predict for lack of response to anti PD-1 therapy [3, 5]. The use of HD IL-2 therapy following immune checkpoint blockade, particularly the impact on therapeutic response and potential toxicity, needs to be investigated further in the context of the rapidly changing field of immuno-oncology. There have been prior reports of colitis and bowel perforation in patients receiving IL-2 therapy following ipilimumab [8].

In addition, understanding the interaction between immunotherapies will help guide future combinations and sequencing of these agents. In this study we queried the PROCLAIM (Additional file 1) database for patients with advanced malignant melanoma who had received HD IL-2 following treatment with ipilimumab and compared them to patients who had not received prior immune checkpoint blockade.

## Methods

### Patients

PROCLAIM (Additional file 1) is an IL-2 patient database with >40 participating sites consisting of a retrospective melanoma (*n* = 170, locked) and prospective melanoma cohort (*n* >335, on-going). The registry is designed to collect data from community and large academic centers on the use of HD IL-2 in the treatment of metastatic renal cell carcinoma (mRCC) or metastatic melanoma (mM) (Clinicaltrials.gov: NCT 01415167) For this study, the prospective cohort was queried to identify patients treated with HD IL-2 after receiving ipilimumab. Patient characteristics, including age, gender, disease type, number and type of prior therapies, duration of ipilimumab therapy and toxicity associated with therapy are reported.

### Treatment

HD IL-2 was administered per the treating institution’s standard of care as an inpatient regimen, typically utilizing a 600,000 IU/kg or 720,000 IU/kg IV infusion every 8 hours as tolerated up to 14 consecutive doses over 5 days. Patients were readmitted for a second week/cycle of treatment after approximately 9 days off therapy, per the discretion of the treating physician. Two weeks of HD IL-2 therapy constituted one course of treatment.

Ipilimumab was administered to patients either as part of a clinical trial or as standard of care therapy at the FDA approved dose and schedule of 3 mg/kg intravenously every three weeks for up to four doses. Eleven of the patients treated on clinical trial were noted to have received more than 3 months of ipilimumab therapy and thus also received maintenance ipilimumab.

### Response data and adverse events

Toxicities in patients treated with HD IL-2 after receiving ipilimumab were examined and compared to subjects who received HD IL-2 without receiving prior ipilimumab. The average number of IL-2 doses per week is also reported. The number of patients who achieved a complete response (CR), partial responses (PR), stable disease (SD) and progressive disease (PD) as determined by the treating physician using RECIST or modified WHO criteria is reported. Objective response rate (ORR) and median overall survival (OS) are also reported. Response and survival endpoints were measured from the start of HD IL-2 therapy and were compared amongst patients who did and those who did not receive prior ipilimumab.

### Statistical analysis

All statistical analyses were performed using SAS software version 9.4 (SAS Institute, Cary, NC). Patient characteristics, tumor response, and survival status were determined using data as of December 2nd, 2015. Frequency counts and measures of central tendency were performed to provide descriptive statistics; medians were reported with the minimum and maximum values. A chi-squared test was performed for toxicities observed during cycle 1. Kaplan-Meier curves with 95 % confidence intervals (CIs) were used to estimate median overall survival (mOS) (the primary outcome), with the log-rank test to determine significance (*P* < .05). Overall survival time was calculated from the date of first dose of HD IL-2 to either the date of death or date of most recent follow-up. The ORR was calculated from the summation of patients reporting CR + PR divided by the total number of patients. The relationship between ipilimumab response and HD IL-2 response was examined with a Fisher Exact test.

## Results

### Patient characteristics

A total of 328 patients were identified within the registry that fit the search criteria. 276 metastatic melanoma patients were treated with HD IL-2 without prior immune checkpoint blockade. Fifty-two metastatic melanoma patients were treated with ipilimumab prior to IL-2. Patients in both groups were predominantly male with ECOG performance status of O or 1 and had greater than or equal to 3 metastases (Table 1). Prior immunotherapy (predominantly adjuvant interferon) was noted in 31 % of those patients that were not treated with prior immune checkpoint blockade. LDH levels prior to initiating HD IL-2 were equivalent between the two groups, mean 291.1 in the prior ipilimumab group and 286.6 in the group without prior immune checkpoint blockade.

**Table 1:** Baseline characteristics

		Prior Ipi only (*n* = 52)		No prior ICB (*n* = 276)		Total (*n* = 328)	
Sex	Female	20	38 %	104	38 %	124	38 %
	Male	32	62 %	172	62 %	204	62 %
Age	<65	43	83 %	234	85 %	277	84 %
	≥65	9	17 %	42	15 %	51	16 %
	Median	52		53		53	
ECOG	0	35	67 %	190	69 %	225	69 %
	1	15	29 %	78	29 %	98	28 %
	2	0		3	1 %	3	1 %
	Missing	2	4 %	5	1 %	7	2 %
# of metastasis	1	13	25 %	82	30 %	95	29 %
	2	20	38 %	92	33 %	112	34 %
	≥3	17	33 %	79	29 %	96	29 %
	Missing	2	4 %	23	8 %	25	8 %
Prior treatments	Surgery	42	81 %	203	74 %	245	75 %
	Radiation	20	39 %	84	31 %	104	32 %
	Chemotherapy	11	21 %	35	13 %	16	5 %
	Immunotherapy	52	100 %	85	31 %	137	42 %
	Targeted therapy	9	17 %	7	3 %	46	14 %
	Other	2	4 %	4	1 %	6	2 %
	No Prior Tx	0		36	13 %	36	11 %
Melanoma Subtype	Cutaneous	36	69 %	195	71 %	231	70 %
	Mucosal	4	7 %	15	5 %	19	6 %
	Ocular	3	5 %	6	2 %	9	3 %
	Acral	1	2 %	5	2 %	6	2 %
	Unknown	8	15 %	55	20 %	63	19 %
BRAF status^a^	WT	20	56 %	84	42 %	104	45 %
	Mutated	16	44 %	114	57 %	130	54 %
	Not done	0		2	1 %	2	1 %
NRAS^a^	WT	10	28 %	64	32 %	74	31 %
	Mutated	6	17 %	12	6 %	18	8 %
	Not done	20	56 %	124	62 %	144	61 %
cKIT^a^	WT	16	44 %	84	42 %	103	43 %
	Mutated	0		8	4 %	8	4 %
	Not done	20	56 %	105	53 %	125	53 %

^a^Mutation status and percentages reported for those patients in whom any mutational testing was reported. For some of these patients only a single genetic test was performed and these patients are included in the chart but listed as having that particular test not done

Mutational status was reported for 36 of the prior ipilimumab only patients and 200 of the patients who had not received prior immune checkpoint blockade. Among those patients in whom mutational status was reported 44 and 57 %, respectively, were noted to be BRAF mutant in the ipilimumab and no ICB groups, 17 % and 6 %, respectively, were noted to be NRAS mutant. Of the patients in who mutational status was tested there were no cKIT mutations noted among the patients in the Ipilimumab group and 8 patients with cKIT mutations in the no prior ICB group.

In the 52 patients who received ipilimumab before HD IL-2, ipilimumab was the therapy that immediately preceded HD IL-2 in 38 of the patients. The average time between completing ipilimumab and starting HD IL-2 was 7.61 months with a range of 1-30.6 months for all patients previously treated with ipilimumab. For those patients in whom ipilimumab was the therapy immediate preceding HD IL-2 therapy; the mean time to HD IL-2 was 6.0 months with a range of 0.4-30 months. On average patients were on ipilimumab for 3.0 months with a range of 0.03-25 months prior to stopping ipilimumab. All patients had progression of their disease prior to initiating treatment with HD IL-2.

The mean time between diagnosis of metastatic disease and initiation of HD IL-2 was 18 months (0.2-80) in patients previously treated with ipilimumab and 7 months (0.03-98) in patients with no prior ICB. The average drug administration duration for HD IL-2 (including rest periods) was 2.0 months for the patients previously treated with ipilimumab and 1.9 months in the patients who were not treated with prior ICB, with the majority of patients receiving only 2 cycles (1 course) of HD IL-2. In the ipilimumab treated group 62 % of patients received 2 cycles and 21 % received four cycles. In the group with no prior ICB 56 % received 2 cycles and 24 % received 4 cycles.

### Toxicity

Adverse events resulting in cessation of HD IL-2 for cycle 1 of treatment are reported in Table 2. The database captured up to 3 toxicites per patient. The pattern of IL-2 toxicity between the groups was relatively similar with cardiac, renal, neurologic and GI toxicities being the most frequently reported. During cycle 1 there was no statistically significant difference observed between the two groups (*p*-value = 1). Of note there were more pulmonary toxicites reported in the prior Ipilimumab group but this difference was not statistically significant. The cardiac toxicities included hypotension, tachycardia, arrhythmia, myocarditis, hypertension and fluid overload.

**Table 2:** HD IL-2 dose limiting toxicities reported for cycle 1 of treatment

	Ipi only (*n* = 52)		No prior ICB (*n* = 276)		Total (*n* = 328)	
Cardiac	13	18.57	84	24.56	97	23.33
Renal	12	17.14	51	14.91	63	15.29
Neurologic	5	7.14	52	15.2	57	13.83
Gastrointestinal	10	14.29	36	10.53	46	11.17
Hematologic	7	10	34	9.94	41	9.76
Metabolic	5	7.14	30	8.77	41	9.76
Pulmonary	10	14.29	19	5.6	29	7.38
Capillary Leak Syndrome	2	2.86	17	4.97	19	4.52
Hepatic	2	2.86	13	3.8	15	3.57
Skin	4	5.71	6	1.75	10	2.38
Total	70	100	342	100	412	100.0

The first column represents the number of times a toxicity was reported among the entire cohort of patients. The second is the percentage of each individual toxicity as compared to the total number of events. The total events add up to more than the number of patients as some patients reported more than one dose limiting toxicity, up to a maximum of three

The average number of HD IL-2 doses per cycle is often used as a reflection of toxicity since IL-2 is held when a patient is having more severe side effects. While this can be influenced by the institution’s standard practice, the fact that similar dose numbers were reached in the two groups suggests similar toxicity profiles. The average number of HD IL-2 doses per cycle is summarized in Table 3.

**Table 3:** Number of doses received during each cycle of HD IL-2

Cycle	Group	*n*	Mean	Min	Max
1	Prior Ipi	52	9.9	3	14
	No ICB	276	9.9	1	14
2	Prior Ipi	49	8.2	1	14
	No ICB	252	8.2	2	14
3	Prior Ipi	17	8.2	5	12
	No ICB	98	8.6	1	14
4	Prior Ipi	17	6.7	2	12
	No ICB	88	6.6	2	14

Institutions involved in PROCLAIM (Additional file 1) reported several patients with severe HD IL-2 toxicity that was considered possibly related to prior ipilimumab. In one case a patient developed increased bloody diarrhea after completing IL-2 treatment. Colonoscopy with biopsy revealed diffuse active colitis with moderate inflammation in the mid sigmoid colon consistent with CTLA4 antibody-related colitis. The patient was started on high dose steroids with resolution of symptoms. Another patient developed severe diarrhea during treatment with HD IL-2 which was resistant to high dose steroid administration. This patient subsequently developed ischemic colitis, acidosis anddied. Neither patient had evidence of CTLA-4 antibody-related colitis prior to starting HD IL-2 therapy.

Within the no prior ICB cohort there were two patients who died during their HD IL-2 therapy. One patient developed altered mental status felt to be related to undetected brain metastasis with subsequent respiratory failure. The second patient developed respiratory failure of unclear origin. The database also looks at prolongation of hospitalization, disability or permanent damage during HD IL-2 therapy. This was reported in 4 of the patients treated with prior ipilimumab (11 %) and 8 of the patients with no prior ICB (4 %) during cycle 1. The toxicities observed in these patients were consistent with those observed with HD IL-2 alone. Within the database only two ipilimumab treated patients were reported as having autoimmune toxicity during IL-2 dosing, one with hepatitis and one with myasthenia gravis. On further investigation these side effects were determined to be HD IL-2 related and not due to recurrence of ipilimumab toxicity.

At the post treatment follow up visits evidence of autoimmune disease was assessed. In the group of patients treated with ipilimumab prior to HD IL-2 6 patients (11 %) reported evidence or autoimmune disease. Of these six patients, 2 had vitiligo, 2 had autoimmune dermatitis, 1 had hemolytic anemia and 1 had autoimmune hepatitis. In the group of patients who received no prior immune checkpoint blockade 16 patients (6 %) reported evidence of autoimmune disease. Of these patients, 5 had thyroid abnormalities, 3 had colitis, 2 had vitiligo and individual patients reported dermatitis, hepatitis, encephalopathy, Guillain-Barre, Myasthenia Gravis and neuropathy. These toxicities were reported at follow up visits and could have been associated with subsequent therapies. Among the patients treated with ipilimumab prior to HD IL-2, 19 had subsequent ICB after HD IL-2, 15 patients had a-PD1 therapy and four patients had combination ipilimumab and a-PD1 therapy. Among the patients who had not received ICB prior to HD IL-2, 138 patients had subsequent ICB after HD IL-2, 84 of these patients received ipilimumab alone, 45 patients received a-PD1 therapy alone and 9 patients received combination ipilimumab and a-PD1 therapy.

### Response rates and survival

The median follow up of all patients was 22 months. Of the patients included in this analysis an overall response rate of 14 % was observed with 10 of the 328 (3 %) patients experiencing a complete response (CR) and 35 (10.7 %) experiencing a partial response (PR). When analyzed by prior treatment group the overall response rate was 21 % in patients previously treated with ipilimumab with 1 (1.9 %) of the patients experiencing a CR and 10 of the 52 (19.2 %) patients experiencing a PR. In the group that had not received prior ICB the overall response rate was 12 % with 9 of the 276 (3.3 %) experiencing a CR and 25 (9.1 %) experiencing a PR. Response data is summarized in Table 4.

**Table 4:** Tumor response to HD IL-2

	Prior Ipi only *n* = 52		No prior ICB *n* = 276		Total *n* = 328	
	*n*	*%*	*n*	*%*	*n*	*%*
*CR*	1	1.92	9	3.26	10	3.05
*PR*	10	19.23	25	9.06	35	10.67
*SD*	15	28.85	74	26.81	89	27.13
*PD*	23	44.23	158	57.25	181	55.18
*Missing*	3	5.77	10	3.62	13	3.96
*Total*	52	100	276	100	328	100
	*n*	%	*n*	%	*n*	%
CR + PR	11	21.15	34	12.32	45	13.72
CR + PR + SD	26	50	108	39.13	134	40.85

**Fig. 1:**
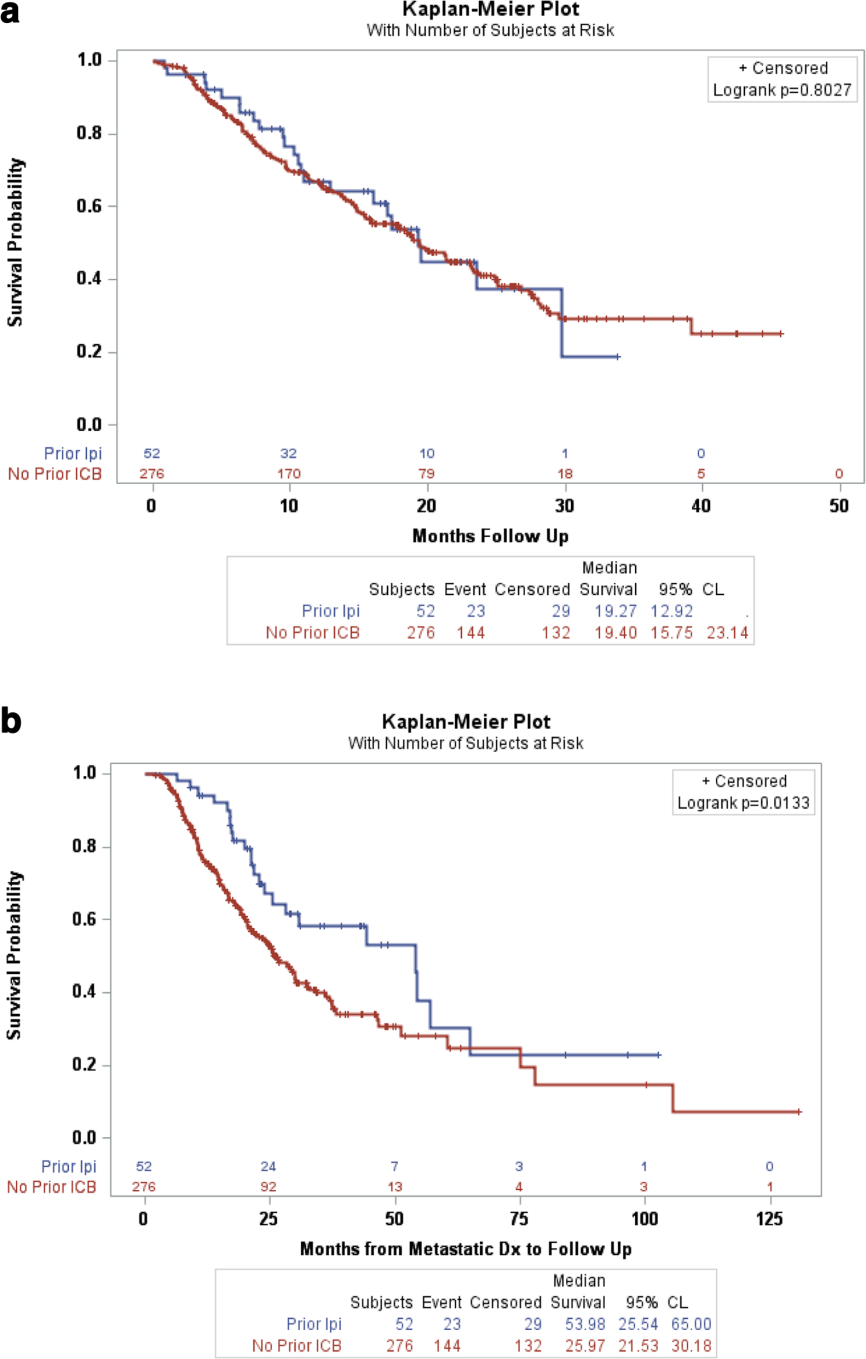
**a** Overall survival after treatment with HD IL-2 therapy in patients treated with prior Ipilimumab as compared to those patients not treated with prior ICB. **b** Overall survival from the time of diagnosis with metastatic melanoma in patients treated with ipilimumab prior to HD IL-2 as compared to those patients not treated with prior ICB

Overall survival, measured from the initiation of HD IL-2 administration, in the group treated with ipilimumab as compared to those patients who had not been treated with prior ICB was equivalent. The mean overall survival was 19.3 months (95 % CI: 12.9, NE) in patients who had received prior ipilimumab as compared to 19.4 months (95 % CI: 15.8, 23.1) in those who had not received prior ICB (*p*-value 0.8027). Kaplan-Meier survival curves are shown for MM both groups in Fig. 1.

Among the patients treated with ipilimumab prior to HD IL-2 one patient had a PR to ipilimumab, 6 patients had stable disease (SD) on ipilimumab, 5 patients are missing data and the remainder of patients had progressive disease (PD). The patient with a prior PR to ipilimumab had SD on HD IL-2, those with prior SD on ipilimumab had outcomes that were varied with one PR, one SD and four having PD on HD IL-2. There was no statistically significant relationship between response to ipilimumab and response to HD IL-2 (*p*-value = 0.6612).

## Discussion

Preliminary retrospective analysis in patients with advanced malignant melanoma suggests that HD IL-2 is a safe treatment option for patients who have had progressive disease after ipilimumab. The overall response rates and survival benefit of HD IL-2 appears were not statistically different when comparing patients with prior ipilimumab and those treated front line with HD IL-2 with ongoing durable responses. In addition overall survival was similar in the two groups despite the fact that patients who had previously received ipilimumab were further into their melanoma treatment, 18 months from diagnosis of metastatic disease as compared to 7 months in the no prior ICB patients. It is possible that some of this effect is due to delayed response to ipilimumab or a synergistic immune response.

High dose IL-2 therapy has predictable toxicity and must be administered in an experienced inpatient setting. In this analysis emergence of CTLA4 antibody induced colitis in patients treated with HD IL-2 following ipilimumab was seen, consistent with a prior report from the National Cancer Institute [8]. However, by the database numbers there was no increase in the overall number of toxicities during HD IL-2 therapy when comparing patients who have received HD IL-2 front line versus those receiving it after ICB. We did not observe immune-mediated hypophysitis in this population, which has been reported in up to 4 % of patients receiving ipilimumab monotherapy. In addition, the number of doses patients received was similar between the two patient populations suggesting that toxicity during HD IL-2 therapy was not significantly altered by prior ipilimumab exposure.

The benefit of HD IL-2 therapy is that the side effects are dose dependent, largely predictable, easily managed and reversible. IL-2-related mortality is less than 1 % at experienced treatment centers with more extensive cardiac screening and better patient selection [7]. However, physicians administering HD IL-2 following ipilimumab should be aware of possible anti-CTLA4- related colitis exacerbation following HD IL-2 therapy since diarrhea is a common symptom with both HD IL-2 and ipilimumab treatment. Patients who had evidence of significant prior CTLA-4 related colitis should only be treated with HD IL-2 after endoscopic evaluation to ensure resolution of colitis. In addition, patients who develop persistent (ongoing or increasing after IL-2 dosing) or bloody diarrhea should undergo diagnostic studies, including stool cultures, CT and/or colonoscopy, to rule out other causes and evaluate for immune-mediated colitis. In addition physicians should consider early use of systemic corticosteroids or other immunosuppressants to control diarrhea and avoid colon perforation or death when immune-mediated colitis is suspected in this clinical setting.

In addition to the potential use of HD IL-2 therapy following immune checkpoint blockade there is also the potential for HD IL-2 to be combined with immune checkpoint blockade. In a small phase Ib clinical trial evaluating the combination of HD IL-2 and dose-escalating ipilimumab, an overall response rate of 22 % was reported with a 8 % complete response rate in a small phase I/II study [9]. A trial of this combination is ongoing and will help determine if diverse immunotherapies have potential synergy (Clinicaltrials.gov: NCT02203604). This analysis is limited to patients treated with ipilimumab prior to HD IL-2 with the plan to examine patients treated with PD1 prior to HD IL-2 as more patients are entered into the database.

There are several limitations of this analysis that may limit its application to daily practice. Data is entered into the database prospectively but this analysis is retrospective and only allows for certain specific data to be captured. The overall numbers of patients remain small and may not capture toxicities observed at institutions which do not participate in PROCLAIM. Investigators could only enter three dose-limiting HD IL-2 related toxicities per patient in an attempt to capture the most severe; however, this may cause an analysis such as this to miss a toxicity that was less severe but more prevalent in the patients previously treated with ipilimumab. Patients were assessed for response by independent investigators using different response criteria and this may have influenced the outcome. Further, ipilimumab has been associated with delayed kinetics of therapeutic response and so it is possible that some patients may continue to exhibit therapeutic anti-tumor activity later in the natural history of their disease. These issues may explain the slightly lower response rate reported overall in this trial compared to historical studies in which HD IL-2 response rates are usually around 16–17 %.

One area of ongoing active investigation includes biomarkers to better predict patients who may benefit from HD IL-2 and immune checkpoint blockade. Numerous studies are evaluating different possible retrospective and prospective markers of immune response [10–13]. As these investigations continue there will be the opportunity to evaluate patients responding to IL-2 post checkpoint blockade to better identify those most likely to benefit. In our trial, there did not appear to any influence on response by tumor mutation status.

## Conclusions

Immune checkpoint blockade is rapidly advancing beyond melanoma into the treatment of renal cell carcinoma, lung cancer, bladder cancer and many other malignancies. As this occurs our need to understand how it potentially interacts with other treatments will be essential to our ability to administer it safely. In addition we continue to require options for patients who do not respond to immune checkpoint blockade, but may be candidates for additional immunotherapy. HD IL-2 remains a viable treatment in melanoma and may be safely administered following progression on ipilimumab.

## Abbreviations

CR, complete response; CTLA4, cytotoxic T-lymphocyte associated protein-4; HD IL-2, high dose interleukin-2; ICB, immune checkpoint blockade; OS, overall survival; PD, progressive disease; PR, partial response; SD, stable disease

## Additional file

Additional file 1: IRB/Human subjects protection offices list for PROCLAIM Registry. (DOCX 10 kb)
