# Supplementary material for: A retrospective analysis of High-Dose Interleukin-2 (HD IL-2) following Ipilimumab in metastatic melanoma
Source: J Immunother Cancer. 2016 Sep 20;4:52. doi: 10.1186/s40425-016-0155-8 (PMC5028986; doi:10.1186/s40425-016-0155-8)
Supplement: Additional file 1: — IRB/Human subjects protection offices list for PROCLAIM Registry. (DOCX 10 kb) [file 40425_2016_155_MOESM1_ESM.docx]

IRB/Human subjects protection offices list for PROCLAIM Registry:

Dana Farber/Harvard Cancer Center IRB

Rutgers Health Sciences IRB

Cleveland Clinic IRB

Providence Medical Research Center IRB

UCSD Human Research Protections Program

MD Anderson Institutional Review Board

University of Pittsburgh Human Research Protection Office

Loyola University IRB

University of Colorado Boulder Human Research and IRB

Saint Louis University IRB

Icahn School of Medicine at Mount Sinai Program for the Protection of Human Subjects

Duke Medicine Institutional Review Board
